# Understanding irritability through the lens of self-regulatory control processes in children and adolescents: a systematic review

**DOI:** 10.1007/s00787-024-02591-8

**Published:** 2024-10-08

**Authors:** Sébastien Urben, Ana Ochoa Williams, Cécile Ben Jemia, Joëlle Rosselet Amoussou, Sara Machado Lazaro, Julia Giovannini, Marion Abi Kheir, Michael Kaess, Kerstin Jessica Plessen, Ines Mürner-Lavanchy

**Affiliations:** 1 Division of Child and Adolescent Psychiatry, Lausanne University Hospital and University of Lausanne, Lausanne, Switzerland; 2 Medical Library-Cery, Site de Cery, Lausanne University Hospital and University of Lausanne, Prilly, Switzerland; 3 University Hospital of Child and Adolescent Psychiatry and Psychotherapy, University of Bern, Bern, Switzerland; 4 Department of Child and Adolescent Psychiatry, Center for Psychosocial Medicine, University Hospital Heidelberg, Heidelberg, Germany; 5 Faculty of Psychology, University of Basel, Basel, Switzerland

**Keywords:** Irritability, Children, Adolescents, Self-regulatory control, Frustration management, Autonomic regulation, Executive function, Effortful control, Parenting, Systematic review

## Abstract

Among youths, pathological irritability is highly prevalent and severely disabling. As a frequent symptom, it often leads to referrals to child and adolescent mental health services. Self-regulatory control (SRC) processes are a set of socio-psycho-physiological processes that allow individuals to adapt to their ever-changing environments. This conceptual framework may enhance the current understanding of the cognitive, emotional, behavioural and social dysregulations underlying irritability. The present systematic review (PROSPERO registration: #CRD42022370390) aims to synthesize existing studies that examine irritability through the lens of SRC processes among youths (< 18 years of age). We conducted a comprehensive literature search among six bibliographic databases: Embase.com, Medline ALL Ovid, APA PsycInfo Ovid, Web of Science Core Collection, the Cochrane Database of Systematic Reviews Wiley and ProQuest Dissertations & Theses A&I. Additional searches were performed using citation tracing strategies. The retrieved reports totalled 2612, of which we included 82 (i.e., articles) from 74 studies. More than 85% of reports were published during the last 6 years, highlighting the topicality of this work. The studies sampled n = 26,764 participants (n = 12,384 girls and n = 12,905 boys, n = 1475 no information) with an average age of 8.08 years (SD = 5.26). The included reports suggest that irritability has an association with lower effortful control, lower cognitive control and delay intolerance. Further, evidence indicates both cross-sectional and longitudinal associations between irritability and a lack of regulation skills for positive and negative emotions, particularly anger. Physiological regulation seems to moderate the association between irritability and psychopathology. Finally, the mutual influence between a child’s irritability and parenting practice has been established in several studies. This review uses the lens of SRC to illustrate the current understanding of irritability in psychopathology, discusses important gaps in the literature, and highlights new avenues for further research.

**Supplementary Information:**

The online version contains supplementary material available at 10.1007/s00787-024-02591-8.

## Background

Irritability is defined as increased proneness to anger in response to frustration relative to peers at the same developmental level [1]. Children who exhibit pathological irritability demonstrate a persistently angry, grumpy mood over extended periods (also known as tonic irritability) and frequent, situationally inappropriate temper tantrums that are not aligned with their developmental stage (described as phasic irritability) [2, 3]. Irritability is among the leading symptoms for referrals to child and adolescent mental health services [4–6]. Among youths, the prevalence of irritability ranges from 20–30% under a broad definition [4, 7] to 1–3% for severe chronic forms [8–11]. In the diagnostic and statistical manual of mental disorders (DSM-5), pathological irritability is listed as a primary or associated symptom in nearly every affective and behavioural disorder [12]. Pathological irritability during childhood is profoundly disabling and is linked to long-term negative consequences, such as reduced educational attainment, poor health, increased delinquency, suicidality and a heightened risk of adult depression, anxiety and conduct disorders [1, 7, 13–21]. Furthermore, childhood irritability is hypothesized to be a transdiagnostic marker of psychopathology spanning both externalizing and internalizing dimensions [20, 22–27].

Irritability refers to a specific form of emotional and behavioural dysregulation. While anger is a normative response to frustrative non-reward [28], chronic negative affective responses to non-reward might stem from a deficit in emotion regulation and could eventually lead to pathological irritability [20]. Self-regulatory control (SRC) processes offer a conceptual framework for understanding these (dys)regulatory mechanisms. SRC includes any intrinsic socio-psycho-physiological process that allows an individual to adapt their cognition, emotions and behaviours to the ever-changing environment or to long-term goals [29]. This may include psychological processes, such as effortful cognitive or executive functions (EF), as well as emotion regulation processes. Moreover, SRC encompasses central (i.e., neural correlates) and peripheral (e.g., heart rate variability [HRV], respiratory sinus arrhythmia [RSA], cortisol) physiological regulation and, finally, social processes (e.g., parenting behaviours or co-regulation) [29].

The current body of literature on the relationship between SRC and irritability offers intriguing insights but remains fragmented and lacks systematic integration. Previous research has established associations between deficits in SRC and increased irritability in children and adolescents [30,31].Indeed, while individual components of SRC—such as inhibitory control, emotion regulation, and cognitive control—have been linked to irritability [30,31], the collective integration of these components and their combined influence on the onset and persistence of irritability have not been comprehensively examined. Moreover, the underlying mechanisms driving these associations, as well as the causal pathways involved, are not yet fully elucidated. A systematic review is therefore essential to synthesize existing findings, identify research gaps, and develop a more holistic understanding of how various SRC processes contribute to irritability. Clarifying the role of these processes is crucial, as it may inform the refinement of assessments, diagnostic criteria, and the development of targeted interventions aimed at improving SRC to mitigate irritability and its associated negative outcomes. By consolidating current knowledge and pinpointing critical research gaps, a systematic review in this area could significantly advance our understanding of the complex interplay between SRC mechanisms and irritability.

### The current systematic review

The aim of this systematic review is to synthesize and assess existing research on the connection between socio-psycho-physiological SRC processes and irritability during childhood and adolescence. In particular, we aim to systematically review existing studies that investigate irritability through the lens of at least one of the SRC processes. This will serve to identify gaps in the present literature and highlight opportunities for future research. By identifying SRC processes that are associated with irritability, this systematic review may provide knowledge to serve as a basis for determining preventive or therapeutic approaches to address irritability.

## Methods

### Procedure

The JBI Manual for Evidence Synthesis, chapter 7: systematic reviews of etiology and risk [32] guided the realization of the review. Also, the preferred reporting items for systematic review and meta-analysis (PRISMA) 2020 [33, 34] were followed for reporting. The review protocol is available on PROSPERO (#CRD42022370390). We incorporated studies that (a) sampled children and adolescents (0–17 years of age); (b) assessed at least one psychophysiological self-regulatory process (e.g., cognitive control, emotion regulation, autonomic regulation or social regulation); (c) measured irritability (e.g., anger proneness, low tolerance to frustration, outbursts); and (d) were published in English, German or French. All study designs were included (i.e., observational and case studies as well as qualitative and quantitative methodology). We excluded studies that (a) were not peer-reviewed or referred to conference acts, (b) focused on psychometric properties of instruments assessing irritability, (c) reported interventions or (d) mainly focused on children and adolescents with an autism spectrum disorder or intellectual disabilities as it refers to neurodevelopmental disorders which imply different developmental pathways, underlying mechanisms and specific assessments [35,36]. Consequently, the SRC processes and their interactions with irritability in these populations may differ significantly from those observed in more typical developmental contexts. Including studies on these populations in the review may therefore introduce excessive heterogeneity, potentially complicating the synthesis of findings and the identification of broader patterns.

Two blind and independent reviewers (SU and AOW) conducted study selection (abstract and title screening as well as full text selection) and data extraction. Choices that differed between reviewers were discussed to achieve a consensus.

### Search strategy

In collaboration with a medical librarian (JRA), a literature search was conducted in October 2023 in six bibliographic databases: Embase.com, Medline ALL Ovid, APA PsycInfo Ovid, Web of Science Core Collection, the Cochrane Database of Systematic Reviews Wiley and ProQuest Dissertations & Theses A&I. The searches were performed without language or date restrictions. Manually (backward search) and through the use of Web of Science Core Collection (forward search), further records were discovered by tracing citations of studies that were included.

**Fig. 1:**
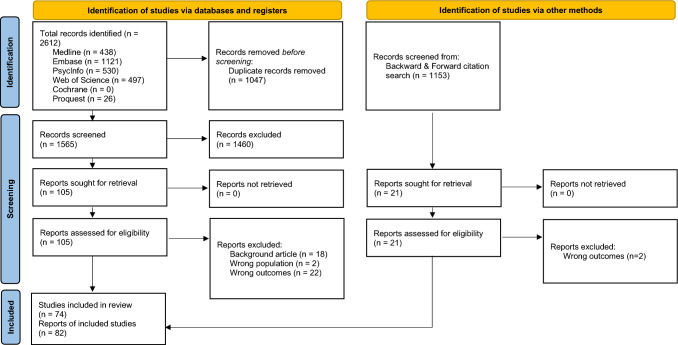
PRISMA 2020 flow diagram for new systematic reviews which included searches of databases, registers and other sources. From: Page MJ, McKenzie JE, Bossuyt PM, Boutron I, Hoffmann TC, Mulrow CD, et al. The PRISMA 2020 statement: an updated guideline for reporting systematic reviews. BMJ 2021;372:n71. https://doi.org/10.1136/bmj.n71. For more information, visit: http://www.prisma-statement.org/

Figure 1 displays the PRISMA 2020 flow diagram [33]. The supplementary File 1 provides details regarding the search syntax, keywords and index terms used.

We screened the titles and abstracts of identified studies for possible inclusion (k = 2612), which led to k = 106 studies selected for full text screening. Among them, 74 studies (and 82 reports) met the inclusion criteria defined in the study protocol (see Table S1). Below, we indicate whether we described the studies (i.e., general project) or the reports (i.e., published article).

### Critical appraisal

We chose the appraisal tool for cross-sectional studies (AXIS) [37], as it seems to be the most appropriate tool for analysing the quality of observational studies. Specifically, the AXIS is a 20-item instrument and was applied by SU and AOW to evaluate the quality of the retrieved reports by assessing the main bias that may be observed in observation studies (e.g., selection of the participants, sample size justification, drop out analyses, statistical method, ethical consideration and role of funding sources; for details, see Table S2 and Figure S2). However, it should be noted that this instrument lacks, for example, aspects such as study protocol and planned analysis publication, blinded analyses or non-published results.

### Study categorization

Number of participants (for both males and females), sample origin and study design were extracted from the retrieved studies. Table 1 describes how irritability was operationalized (e.g., frustration, anger proneness, aggressivity, anger dysregulation, grumpy mood, tantrum). Finally, we characterized the specific process of SRC (i.e., cognition [either “purely” or combined with “affective/motivation” dimensions], emotion, physiology and social) for each study (see Figure S1 for overlaps between the studies regarding which processes were examined).

**Table 1:** Terms used to describe irritability

Processes	Terms	k	%
Irritability (phenotype)	Both	66	80.5
	Phasic	7	8.5
	Tonic	9	10.9
Irritability^a^ (discrete expression, manifestation)	Irritabilty general	66	69.5
	Anger proneness	8	8.4
	Frustration intolerance	7	7.4
	Temper tantrum	8	8.4
	Aggression	5	5.3
	Grumpy mood	1	1.1

^a^More than one per article possible

## Results

### Study description

The included reports were published between 1981 and 2023. However, only three reports were published before 2000, and k = 70 reports (85.4%) were published after 2017 (within the last 6 years), from which k = 14 reports (17.1%) were published in 2023, highlighting the topicality of this subject. The vast majority of studies were performed in Western countries, particularly in the United States (k = 48, 64.8%) and in European countries (k = 18, 24.3%). Less than 7% of the studies (k = 5) originated from Asia and South America. Most studies adopted a cross-sectional design (k = 31, 41.3%). Further, k = 29 studies (39.1%) and k = 14 studies (18.9%) adopted a longitudinal design (with follow-up ranging from 6 months to 17 years) and a case–control design, respectively. A total number of n = 26,764 participants (n = 12,384 girls and n = 12,905 boys, n = 1475 no information) were present in the selected studies. The study samples referred mainly to community samples (k = 42, 56.8%) or clinical samples (k = 32, 43.2%). In thirteen studies, more than 60% of those sampled were boys [38–46], whereas in two studies, more than 75% of those sampled were boys [47, 48]. In four studies, more than 60% of those sampled were girls [49–52], whereas one study sampled girls, exclusively [52]. Finally, four studies did not specify the gender in their samples [31, 53–56]. The remaining reports included an equivalent proportion of boys and girls. The average age of all who were sampled was 8.08 years (SD = 5.26). Children younger than 5 years old were included in k = 29 studies (39.2%), and 26 studies (35.1%) sampled adolescents above 12 years old. Only parents were included in the samples of twenty-one studies (28.4%), from which k = 14 recruited only mothers and k = 7 included both mothers and fathers. The majority of reports assessed both tonic and phasic irritability components (80.5%) without distinguishing them. The reason for doing so is that the instruments did not allow for a distinction to be made between components. For terminology, the majority of reports (69.5%) used “irritability” without specification. In 8.4% of reports, “tantrum” was used alongside other terms such as anger proneness, frustration intolerance, aggression or grumpy mood.

Regarding the SRC components, approximately one quarter (25.7%) of the reports examined physiological aspects. Conversely, either “purely” cognitive aspects or “affective/motivation” combined with cognitive aspects were examined in about 20.8% of reports. Finally, emotional components (i.e., emotion regulation) were considered in almost 14% of the reports.

### Critical appraisal

The AXIS assessment for each specific report is presented in the supplement (see supplementary Table S2 and Figure S2). Only k = 2 (2.4%) reports justified the sample size, either by a priori or a posteriori power analysis. Moreover, the question of drop out was addressed in k = 13 (15.9%) studies, of which seven reports described drop out bias (the vast majority of reports [k = 66; 79.5%] did not address this aspect). Patients with the most severe forms of irritability might have been more prone to drop out. Therefore, important information on the full range of irritability might have been overlooked by these studies. Overall, the included studies were of high quality, with none demonstrating a particularly strong bias, as measured with this instrument.

### Results summary—narrative review

**Fig. 2:**
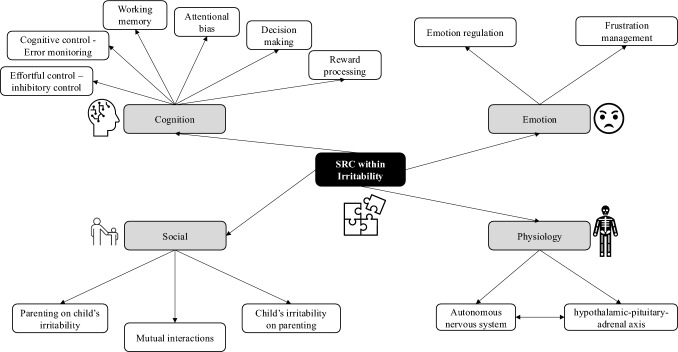
Thematic organization of the included reports. *SRC* self-regulatory control processes

In the following subsections, we provide a narrative summary of the included studies according to the perspectives of the main SRC processes (cognitive, emotional, physiological and social) on irritability. We illustrate the main themes and subthemes examined in the included reports (Fig. 2). Also, we highlight the main characteristics and findings for each report (Table S1).

#### Cognitive SRC

One of the main findings of the reports was that during early life, a protective role of effortful control and inhibitory control regarding “purely” cognitive (or non-affective) aspects of SRC was present [31, 51, 57–60, 144]. One study observed stronger relationships between inhibitory control and, later, irritability in girls than in boys [59]. From middle childhood, cognitive control (i.e., more elaborated inhibitory skills such as error monitoring) was cross-sectionally associated with [42, 61, 62] and longitudinally predicted [54, 63–65] lower irritability in adolescence. Moreover, the combination of low cognitive control and high irritability was a risk factor for concurrent and, later, internalizing and externalizing problems (e.g., [31, 66]). When combined with neuroimaging or psychophysiological assessment of cognitive control, irritability was related to aberrant brain functioning (i.e., error-related negativity [63] or less neural activation in the left dorsolateral prefrontal cortex [62]) as well as increased HR and decreased HRV (not differing in function of gender) [46]. Other reports identified both the protective role of working memory in the associations between irritability and psychopathology [67, 68] as well as the mediating role of irritability in low executive functions and aggression, which was similar across genders [69].

Another group of studies [70–75, 145] revealed that higher irritability was related to higher attentional bias in the processing of emotional information. Particularly, children and adolescents with irritability showed enhanced attention towards threat [72] or anger [70], which has been observed both at the cognitive level and through alterations of the neural activation that sustains these processes. Such attentional bias was observed in associations with irritability but not with callous-unemotional traits [73].

A further cognitive domain associated with irritability is temporal reward discounting (i.e., choosing smaller immediate rather than larger delayed rewards), with higher discounting associated with higher irritability both at behavioural [38] and neural [49, 76] levels across development. Preliminary findings suggested that cognitive flexibility and inhibitory control may buffer irritability-related reward processing deficits [74]. Interestingly, irritability had no impact on decision-making [44, 45].

#### Emotional SRC

Both cross-sectionally and longitudinally, irritability has been associated with a lack of emotion regulation skills for positive [77] and negative emotions [55, 78, 79, 149], especially anger regulation [80]. Irritability has been identified as a risk factor for externalizing problems (via poor sadness/anger regulation), oppositionality and internalizing symptoms (via poor anger coping and intolerance to uncertainty) [81]. More specifically, youths with higher irritability in lab-based frustrative situations (in-situ approach) displayed higher emotional arousal as well as slower recovery at behavioural [82, 150], neural [83–90, 146, 148] or autonomous [41, 91] levels. In this line, lower neural parent–child synchrony during frustration recovery was associated with higher child irritability [92]. School-aged children with chronic irritability were characterized by frustration management difficulties as well as inhibitory control deficits [93]. Further, in the context of frustration, autonomic inflexibility (low RSA, i.e., coupling between heart rhythm and respiration) combined with deficits in inhibitory control sustained irritability in children [94]. Finally, irritability but not anxiety (i.e., lack of regulation of fear) was associated with dysfunctional processing of emotional stimuli. However, both irritability and anxiety disrupt emotion regulation [95]. Moreover, anxiety is closely related with irritability, at least at the neural level [74, 75].

#### Physiological SRC

Although irritability has not been associated with higher hair cortisol levels [39], different diurnal cortisol patterns mediate the link between irritability and, later, psychopathology [53]. In newborns, higher HR was associated with higher irritability, whereas irritability was not consistently associated with cortisol response to the Neonatal Behavioral Assessment Scale [96].

#### Social SRC

The last group of reports examined, mainly through parent–child interactions, links between childhood irritability and the social aspects of SRC (co-regulation). Notably, negative parenting [145] (e.g., low maternal sensitivity [97–99], low maternal social support [100], maternal emotion regulation difficulties [101], harsh parenting [102] or authoritative practices [103]) were associated with higher irritability in children or adolescents. Conversely, higher irritability in children was associated with intrusive and less physically stimulating maternal behaviours [104] and led to more maternal coerciveness [105] or higher negative parental attitudes [43]. In this line, toddlers with highly stable profiles between 30 and 42 months of age with “*expressive*” profiles (i.e., higher anger proneness and activity) received less positive parenting and presented more externalizing symptoms. In contrast, “*fearful*” profiles (i.e., higher anger proneness and social fear) received less positive and more negative parenting and presented more internalizing problems. This pattern did not differ in function of gender [106]. These apparent bidirectional associations may further lead to vicious circles. For instance, more irritability may trigger negative parenting (e.g., punishment), which, in turn, leads to more irritability later in development [56]. Moreover, the transmission of maternal internalizing symptoms (when the child is 3 y.o.) to the child’s internalizing symptoms at 11 y.o. is mediated by child irritability at 8 y.o. [107]. The combination of higher irritability (i.e., anger proneness) and less authoritarian parenting has been observed to have a negative impact on cognitive ability, which affects both genders equivalently [108].

In longitudinal studies, parenting modulated both the relationship between irritability and effortful control and the development of adjustment problems during the transition to adolescence [109]. Specifically, when children are assessed at age 3, both components (i.e., phasic and tonic) are observable and distinguishable. Phasic irritability independently was concurrently associated with lower effortful control and higher maladaptive parenting, whereas tonic irritability independently predicted disruptive and suicidal behaviours in adolescence [110]. None of the studies considered the role of siblings or peers in the manifestation of irritability, which may be of particular interest given the importance of peer interactions for children and adolescents.

## Discussion

This systematic review aimed to synthesize the existing literature on cognitive, emotional, physiological and social SRC processes and irritability across the development of children and adolescents. We identified 74 studies (and 82 reports), most of which were published after 2017, emphasizing the topicality of this review. Cognitive processes linked to heightened irritability included low cognitive control, poor delay discounting and a bias toward threat, while emotional SRC showed poor emotion (especially anger) regulation as well as higher emotional arousal. Few studies in the psychophysiological domain suggest changes in endocrinological and autonomic functioning related to high irritability. Finally, social SRC revealed bidirectional associations between higher irritability and parenting difficulties. The systematic search revealed several important findings and research gaps. In the following section, we will discuss these gaps and integrate our reasoning that suggests developmental pathways of SRC processes in irritability.

### Integration of findings and gaps

One of the most clinically relevant aspects of the research on SRC and irritability is how the associations between them unfold over the long-term, especially from infancy to childhood, adolescence and young adulthood. Notably, knowledge on the developmental pathways of irritability may guide prevention and intervention strategies. Further, the manner in which these associations predict clinical outcomes, such as internalizing or externalizing psychopathology, is of crucial importance. A considerable number of identified studies have used a longitudinal design, but few have focused on irritability and SRC as predictors of later psychopathology [31, 53, 58, 59, 67, 69, 81, 106, 107, 110–112], and very few have used clinical samples [67, 112].

Establishing comparability due to variations in both the definition and measurement of the construct of irritability is one challenge across the studies identified in this review [113]. Recent research has reached a consensus on defining irritability as an increased proneness to anger that may lead to aggression but often does not [20, 114, 115]. Anger is described as a transient negative emotional state ranging from mild irritation to intense rage, with physiological, cognitive and behavioural components. Aggression refers to intentional behaviours that cause harm, including reactive, impulsive or proactive types. However, distinguishing between irritability, anger and aggression proves challenging, as the constructs are closely intertwined and because rating scale measures of the constructs correlate with medium to large effect sizes [116–118], raising doubts about their distinctiveness. The reliability of assessments of irritability (as well as the other constructs) is further complicated by differences in informants, with small to medium correlations between reports from different sources [80, 116, 118]. It is likely that irritability manifests differently at different ages as well as to various degrees in different contexts (e.g., at home vs. in school), and informant perspectives differ not just due to measurement error. In the studies, emotional SRC has primarily been analysed via parent report or physiological measures during challenging situations, offering insight into either the perception that the parent has of their child’s general functioning or real-time observation of emotion regulation reactivity and recovery phases. However, there remains a lack of child and youth self-reporting in this domain.

Few studies have examined peripheral physiological SRC processes such as ANS or HPA-axis functioning in relation to irritability, suggesting a need for more detailed examination in future research. While many reports have outlined aberrant neural patterns associated with irritability or related constructs, findings are mixed, and a recent meta-analysis showed no common functional or structural substrates underlying irritability [119].

In exploring the role of social interaction in regulating irritability, numerous studies have shed light on the reciprocal relationship between irritability and parenting, predominantly within a core family and primarily focusing on mothers. However, limited attention has been given to the social components of SRC outside the family context, particularly in that of peer interactions or support. Considering the importance of peer relationships in middle childhood and especially in adolescence, investigating their influence on irritability would be exceptionally valuable.

A further research gap that has emerged from our systematic review is the role of gender in the association between SRC and irritability, which has been reported in only five studies. We found only one study in which stronger relationships between inhibitory control and, later, irritability were observed in girls than in boys [59]. More generally, this topic has received little attention in research on irritability [120]. However, some evidence from population-based studies suggests that developmental patterns differed between males and females, with irritability being more common during childhood (decreasing with age) in boys and during adolescence (with levels increasing with age) in girls [121, 122]. The authors suggested two “types” of irritability based on gender: an early-onset type more common in boys (a pattern typical of neurodevelopmental problems) and a later-onset “type” that starts in adolescence and is more common in girls (a pattern typical of mood problems) [121]. In this perspective, one longitudinal study reports early differential associations between the sexes and in parental symptoms in predicting phasic and tonic irritability in adolescence [123]. Thus, future studies that investigate SRC processes of irritability should be designed with a specific focus on gender.

**Fig. 3:**
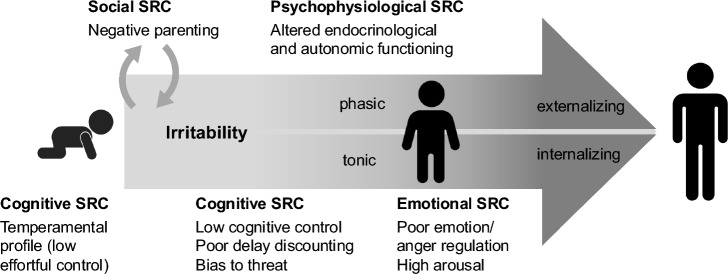
Proposed developmental pathways

The areas that were explored in previous studies and identified in this systematic review delineate the potential developmental pathways of SRC for irritability (see Fig. 3). More specifically, irritability may arise from a problematic temperamental profile. Then, it is increased through delay intolerance, low cognitive control skills and attention bias towards anger or threat. Mutual negative influence is eventually observed through irritability and parenting. Finally, emotion dysregulation and physiological regulation mediate the link between irritability and specific psychopathology. The proposed developmental pathways entail certain gaps that need to be substantiated in future studies.

### Future perspectives

In previous research, irritability was mainly studied as a unitary clinical phenomenon [124, 125]. This resulted in a poor definition of irritability and an unclear understanding of its clinical correlates and pathophysiology. To overcome this shortcoming, tonic and phasic irritability are distinguished in more recent conceptualization [126–128], the usefulness of which should be tested in future studies. Similarly, the majority of studies have focused on single components of SRC. However, in daily life, all SRC processes work together to allow the individual to adapt to the ever-changing environment. While certain studies have incorporated multiple SRC components, a need exists for their integration in future studies.

Moreover, research on irritability may benefit from a more ecological or naturalistic approach. Taking into account both a between-person and a within-person perspective as opposed to the standard or static approach may offer an improvement in the understanding of the nature and variability of irritability over time (e.g., [129–131]). In addition, this approach will allow for an assessment of the temporal sequences among irritability and SRC [132, 133]. Such knowledge is of crucial importance for developing ecological momentary interventions or just-in-time adapted interventions (see [134]). Furthermore, adopting an integrative approach in ambulatory assessment, which involves not only repeated prompting of subjective experiences but also cognitive assessments and physiological indices, may enhance the current understanding of the dynamic interplay between psychological and physiological factors within the everyday contexts that individuals encounter.

The studies included in this review are international in scope; however, the cultural conceptualizations of irritability (and the implication of SRC processes) have not been specifically addressed in prior research or in this systematic review. The perception, expression, and regulation of emotions—such as anger or irritability—vary significantly across cultures [135, 136] , shaped by culturally shared “decoding rules” [137], which influence how individuals interpret and express emotions within their specific cultural context. Moreover, the threshold for what is considered irritable behavior can differ according to cultural norms and expectations [8]. Consequently, cross-cultural studies are essential for examining both the similarities and differences in the phenotyping of irritability and its association with SRC processes. In this context, the Cross-Cultural Consortium on Irritability (C3I, https://medicine.yale.edu/childstudy/research/collaborative-labs/cross-cultural-consortium-irritability/) is a noteworthy collaborative initiative dedicated to investigating irritability across different cultures. The C3I aims to establish an international network of researchers to advance understanding of the cross-cultural similarities and differences in irritability, with a focus on, though not limited to, the pediatric population.

### Clinical relevance and implications

It has been reported that the prevalence of irritability is rising substantially in community samples [4]. Hence, obtaining a better understanding of irritability through the lens of SRC may represent the foundation for intervening early. Childhood represents an early window of opportunity for the prevention of negative long-term outcomes for the affected individuals [18] and might inform the development of therapeutic interventions on specific SRC processes. For instance, cognitive remediation targeting specific self-control deficits [139, 140] and biofeedback [141, 142] or virtual reality techniques [143] targeting specific psychophysiological SRC may represent an interesting therapeutic approach.

### Limitations of this review

We limited this systematic review to children and adolescents. Thus, further reviews should focus on adults. Moreover, this systematic review was limited to literature published in English, German and French. We may, therefore, have missed some information published in other languages. However, this is unlikely to taint the global observed picture, as we did not limit the search based on language in the first place. Due to the heterogeneity in study designs and operationalizations of the associations between irritability and SRC, we did not conduct a quantitative meta-analysis, which will be helpful in the future when methodologies are more harmonious and, thus, comparable.

## Conclusion

Irritability and SRC are closely related. Previous research contained an examination of several SRC components, which we synthesize in the present systematic review. Several challenges and gaps were identified, such as the conceptualization and measurement of irritability, the scarcity of longitudinal studies exploring SRC and irritability as predictors of psychopathology, and the potential of psychophysiological as well as multi-informant and peer relationship studies. Moreover, the current understanding of irritability might benefit from an assessment of its tonic and phasic components, which may be performed with the use of more naturalistic methodologies. The acquired insights might contribute to the development of new therapeutic interventions aimed at alleviating the challenges associated with irritability that youths and their families encounter.

## Supplementary Information

Below is the link to the electronic supplementary material.

Supplementary file1 (DOCX 365 KB)

## Data Availability

No datasets were generated or analysed during the current study.
