# Supplementary material for: Understanding irritability through the lens of self-regulatory control processes in children and adolescents: a systematic review
Source: Eur Child Adolesc Psychiatry. 2024 Oct 8;34(5):1497–509. doi: 10.1007/s00787-024-02591-8 (PMC12122652; doi:10.1007/s00787-024-02591-8)
Supplement: Supplementary file 1 — Supplementary file1 (DOCX 365 KB) [file 787_2024_2591_MOESM1_ESM.docx]

Figure S1. Components of self-regulatory processes examined in the included reports

Cool Cognition

k = 34

Hot Cognition

k = 29

Emotion

k = 20

Physiology

k = 36

Social

k = 23

k = 5

k = 9

k = 7

k = 7

k = 21

k = 9

k = 6

k=2

k=3

k=1

Hot

cognition

Social

k=1

k=3

k=1

Physiology

k=2

Physiology

k=2

k=1

Table S1. Main characteristics of reports

| **Study #** | **1st author & ref** | **Year** | **N** | **Parents** | **Boys** | **Girls** | **Mean age** | **Sample categorization** | **Study design** | **Main findings** |
| --- | --- | --- | --- | --- | --- | --- | --- | --- | --- | --- |
| 1 | Ali [90] | 2023 | 81 | Yes | 45 | 36 | 3.4 | Community | Longitudinal | **↑** irritability at age 3 **→↓** reactivity in the dLPFC related to maternal criticism (more specifically in youths with more positive maternal parenting) |
| 2 | Ametti [94] | 2022 | 294 | No | 197 | 97 | 10.9 | Clinical (ADHD, ODD, GAD, MDD, ODD) | Cross sectional | Autonomic inflexibility (low RSA) in context of frustration + deficits in inhibitory control **↔** dysregulation in children |
| 3 | Armour [105] | 2018 | 1440 | No | 737 | 703 | 0.4 | Community | Longitudinal | infant’s irritability **→** maternal coerciveness (one year later) |
| 4 | Barbosa [98] | 2019 | 121 | Yes | 63 | 58 | 0.3 | Community | Cross sectional | Maternal sensitivity is important for infant behavioral regulatory patterns (including irritable temperament) |
| 5 | Blair [38] | 2020 | 195 | No | 123 | 72 | 16.4 | Clinical (CD) | Case-control | **↓** Temporal reward discounting **↔** risks of irritability |
| 6 | Braenden [144] | 2023 | 208 | No | 126 | 82 | 9.7 | Clinical (DMDD, Depression, Anxiety, ADHD, CD, ODD) | Case-control | **↑** irritability **↔ ↓** emotion regulation and cognitive flexibility |
| 6 | Braenden [39] | 2023 |  |  |  |  |  |  |  | No specific link between cortisol concentrations and irritability |
| 7 | Calkins [104] | 2004 | 162 | Yes | 79 | 83 | 0.5 | Community | Cross sectional | **↓** infant’s frustration **↔** maternal **↓** intrusiveness and **↑** physically stimulating |
| 8 | Cardinale [65] | 2022 | 89 | No | 48 | 41 | 16.3 | Community | Longitudinal | **↑** irritability **↔** **↑** neural activation during a modified Flanker task in regions involved in response inhibition |
| 9 | Cardinale [44] | 2021 | 95 | No | 43 | 52 | 13.0 | Clinical (ADHD, MDD, Anxiety) | Cross sectional | Decision making or willingness to explore ↮ irritability |
| 10 | Cave-Freeman [101] | 2023 | 371 | Yes | 187 | 183 | 2.1 | Community | Cross sectional | Maternal maladaptive emotion regulation assistance to their child **↔** mother’s own emotion regulation difficulties; 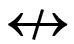 child’s irritability |
| 11 | Cha [108] | 2018 | 80 | Yes | 46 | 34 | 4.9 | Community | Cross sectional | **↑** infant’s irritability + **↓** authoritarian parenting **↔ ↓** performance on a spatial cognitive task |
| 12 | Chaarani [64] | 2020 | 81 | Yes | 45 | 36 | 3.4 | Community | Longitudinal | Transdiagnostic pattern: **↑** irritability **↔** **↓** neural activation underlying successful response inhibition |
| 13 | Colonna [47] | 2022 | 219 | No | 206 | 13 | 14.0 | Clinical (ADHD) | Longitudinal | Irritability ↮ cool as well as hot EF (in youths with ADHD) |
| 14 | Crockenberg [100] | 1981 | 48 | Yes | 25 | 23 | 0.3 | Community | Longitudinal | In mothers with irritable babies: maternal social support **→** future secure attachment (first year of life) |
| 15 | Crum [95] | 2021 | 155 | No | 92 | 63 | 15.3 | Clinical (ADHD, ODD, CD, MDD, GAD, SAD, PTSD) | Cross sectional | Irritability but not anxiety **↔** atypical processing of emotional stimuli; ↑ anxiety + **↑** irritability **↔↓** emotion regulation |
| 16 | Derella [93] | 2023 | 63 | No | 31 | 32 | 9.9 | Community | Case-control | School-age children with chronic irritability: **↓** frustration management + **↓**inhibitory control deficits |
| 17 | Deveney [82] | 2018 | 46 | No | 20 | 26 | 5.4 | Clinical (SMDD) | Cross sectional | ↑ parent-reported temper loss **↔** **↓** behavioral and neural performances during frustration condition in a response inhibition task |
| 18 | Deveney [40] | 2013 | 42 | No | 26 | 16 | 14.0 | Clinical (SMDD) | Case-control | Frustration induction: **↓** activation in brain regions involved in emotion salience, spatial attention, and reward processing, impairing attention flexibility |
| 19 | Di Giunta[102] | 2020 | 1298 | Yes | 636 | 662 | 13.1 | Community | Longitudinal | Highly irritable parents + harsh parenting **↔** adolescents’ symptoms of irritability + internalizing and externalizing problems |
| 20 | Dougherty [49] | 2018 | 46 | No | 18 | 28 | 4.3 | Clinical (Depression) | Longitudinal | Irritability **→** aberrant patterns of brain connectivity related to reward processing |
| 21 | Elvin [66] | 2021 | 93 | No | 52 | 41 | 10.0 | Community | Cross sectional | Three distinct profiles: (a) low irritability and high self-regulation, (b) moderate irritability and low behavioral control, and (c) high irritability and low self-regulation of negative emotions |
| 22 | Evans [81] | 2020 | 238 | No | 124 | 114 | 8.9 | Community | Longitudinal | Irritability (via poor sadness / anger regulation) **→** externalizing problems or oppositionality; Irritability (via poor anger coping and intolerance to uncertainty) **→** internalizing problems |
| 23 | Ezpeleta [58] | 2019 | 614 | Yes | 308 | 316 | 3.8 | Community | Longitudinal | Lower effortful control at age 3 **→** higher irritability at age 4 **→** higher affective problems, anxiety, and oppositional defiant problems at age 7 |
| 24 | Filippi [63] | 2020 | 291 | No | 135 | 156 | 0.3 | Clinical (Social anxiety) | Longitudinal | **↑** irritability **↔ ↓**  ERN, a neural indicator of error monitoring and cognitive control |
| 25 | Giller [71] | 2021 | 120 | No | 67 | 53 | 10.2 | Clinical (Affective dysregulation) | Case-control | Irritability **↔** **↓** differentiating between conflicting and non-conflicting cognitive-emotional information |
| 26 | Grabell [86] | 2018 | 92 | No | 56 | 36 | 5.3 | Clinical (SMDD) | Cross sectional | irritability Ո hemodynamic response (especially in the lateral prefrontal cortex) related to frustration management |
| 27 | Grabell [41] | 2022 | 73 | No | 39 | 34 | 4.6 | Community | Cross sectional | ↑ irritability **↔** functional dysconnectivity between the PFC and the ANS while managing frustration |
| 28 | Gunning [97] | 2013 | 122 | Yes | 58 | 64 | 0.3 | Clinical (Behavioural dysregulation) | Case-control | Maternal insensitivity **↔**  ↑ irritability **↔ ↓** behavioral and physiological regulation when facing challenging situations |
| 29 | Haller [42] | 2021 | 187 | No | 124 | 63 | 13.1 | Clinical (ADHD, DMDD) | Case-control | Cognitive endophenotype for both ADHD and DMDD: **↓** processing efficiency of cognitive demands |
| 30 | Harle [88] | 2022 | 45 | No | 18 | 27 | 15.8 | Community | Cross sectional | Frustration induction: **↓** default mode network (i.e., precuneus) +**↓** regions underlying goal directed reward-based decision making in adolescents (i.e., putamen/caudate and frontoparietal cortex) |
| 31 | Hodgdon [83] | 2021 | 31 | No | 13 | 18 | 14.5 | Community | Cross sectional | ↑ irritability: neural correlates of difficult recovery from frustration (i.e., frustrative non-reward) |
| 32 | Karalunas [67] | 2021 | 488 | No | 312 | 176 | 9.1 | Clinical (ADHD) | Longitudinal | Differential roles of both working memory and temperamental irritability in the development of ADHD and depression; irritability **↔** core symptoms of ADHD and depression |
| 33 | Karalunas [68] | 2023 | 849 | No | 524 | 325 | 9.4 | Clinical (ADHD, ODD, Depression) | Case-control | Irritability + inhibitory control and working memory ↛ anxiety in adolescence;  **↑** working memory + **↑** irritability **→** onset of depression + worse outcomes |
| 34 | Karim [79] | 2017 | 36 | No | 16 | 20 | 7.6 | Community | Cross sectional | Irritability (i.e., temperamental anger/frustration) moderated the developmental changes during childhood in the neural systems sustaining negative emotion regulation |
| 35 | Kessel [53] | 2021 | 541 | No | NA | NA | 3.6 | Community | Longitudinal | **↑** persistent irritability at age 3 **→** internalizing symptoms at age 12 (via steeper diurnal cortisol slope at age 9); → externalizing symptoms at age 12 (via blunted diurnal cortisol slope at age 9) |
| 35 | Kessel [54] | 2016 |  |  |  |  |  |  |  | **↑** ERN during cognitive control at 3 + irritability **→** internalizing disorders at 6 and 9; blunted or **↓** ERN during cognitive control at age 6 **→** externalizing disorders at age 9 |
| 36 | Kryza-Lacombe [76] | 2020 | 52 | No | 24 | 28 | 13.8 | Clinical (Anxiety, Depression) | Cross sectional | Irritability **↔** aberrant patterns of brain connectivity related to reward processing |
| 36 | Kryza-Lacombe [145] | 2020 |  |  |  |  |  |  |  | ↑ irritability ↔ ↑ fluctuation in neural reactivity (↔ anxiety) |
| 36 | Kryza-Lacombe [74] | 2022 |  |  |  |  |  |  |  | EF (i.e., cognitive flexibility and inhibitory control) buffered irritability-related reward processing deficits;  Irritability (more than anxiety) **↔ ↓** neural reactivity related to the impact of emotional faces on attention shifting whereas anxiety symptoms impacted neural correlates underlying irritability |
| 37 | Lee [61] | 2022 | 5948 | No | 2802 | 3146 | 9.9 | Community | Cross sectional | Neural correlates of response inhibition, but not error processing (in Stop signal task) **↔** ADHD + irritability |
| 38 | Lee [103] | 2013 | 425 | Yes | 189 | 236 | 7.7 | Community | Longitudinal | Parent authoritative practices **→** children: **↓** regulatory and ↑ anger/frustration |
| 39 | Legenbauer [50] | 2018 | 91 | No | 27 | 64 | 14.0 | Clinical (Affective dysregulation) | Case-control | Chronic irritability **↔** depression symptoms and affective dysregulation but not maladaptive emotion regulation strategies |
| 40 | Leigh [78] | 2020 | 165 | No | 94 | 71 | 13.2 | Community | Longitudinal | ↑ anger rumination **→** ↑ irritability |
| 41 | Lengua [111] | 2003 | 190 | Yes | 85 | 105 | 9.5 | Community | Longitudinal | Irritability + attention regulation, inhibitory control, and impulsivity **→** internalizing and externalizing problems |
| 41 | Lengua [109] | 2006 |  |  |  |  |  |  |  | Parenting modulated the relationship between irritability and effortful control, and the development of adjustment problems |
| 42 | Li [62] | 2017 | 46 | No | 24 | 22 | 4.8 | Community | Cross sectional | Cognitive flexibility + irritability **↔** dLPFC activation |
| 43 | Linke [87] | 2023 | 66 | No | 33 | 33 | 14.0 | Clinical (DMDD, ADHD, ODD, Anxiety) | Case-control | Brain reconfiguration of connectivity **↔** emotional and cognitive demands sustaining frustration management **↔** irritability |
| 44 | Liuzzi [51] | 2020 | 19 | No | 7 | 12 | 13.2 | Community | Cross sectional | **↑** irritability **↔** aberrant brain activation sustaining inhibitory control |
| 45 | Malhi [52] | 2021 | 229 | No | 0 | 229 | 14.6 | Community | Cross sectional | Irritability **↔** depressive symptoms was fully mediated by trait anxiety **↔** emotion regulation, whereas irritability was **↔** manic symptoms through a decrease in impulse control |
| 46 | Morris [146] | 2002 | 40 | Yes | 16 | 24 | 7.6 | Community | Cross sectional | **↑** irritable children + maternal hostility **↔**  externalizing problems;  **↑** irritable children + maternal psychological control **↔** internalizing problems |
| 47 | Naim [46] | 2021 | 51 | No | 32 | 19 | 12.6 | Clinical (ADHD, Anxiety, DMDD) | Cross sectional | Irritability **↔** **↑** HR + **↓** HRV while performing an inhibitory control task (i.e., Stop signal task) |
| 48 | Naim [70] | 2022 | 351 | No | 179 | 172 | 12.9 | Clinical (ADHD, DMDD, Anxiety) | Cross sectional | aberrant neural correlates of attention regulation **↔ ↑** attention bias to angry stimuli |
| 49 | Nili [31] | 2022 | 223 | No | NA | NA | 4.4 | Community | Longitudinal | **↑** irritability + **↓** inhibitory control **↔** internalizing and externalizing symptoms + symptoms of ODD |
| 50 | Nobakht [55] | 2023 | 641 | No | NA | NA | 4.4 | Community | Longitudinal | **↓** emotion regulation **→** **↑** irritability |
| 51 | Perez [48] | 2021 | 106 | Yes | 82 | 24 | 15.4 | Community | Cross sectional | **↑** parental control + **↑** maternal anxiety + **↑** interparental conflict **↔** **↑** adolescent’s distancing, and frustration (or irritability) |
| 52 | Perhamus [59] | 2021 | 300 | No | 168 | 132 | 3.7 | Community | Longitudinal | Deficits in inhibitory control **→** higher irritability **→** more reactive physical and relational aggression |
| 53 | Perlman [147] | 2015 | 54 | No | 31 | 23 | 8.0 | Clinical (Bipolar, Major depression, Anxiety, ADHD, ODD, CD) | Case-control | Irritability **↔** reward and frustration management through neural dysfunction in regions involved in reward processing, error monitoring, and emotion regulation |
| 54 | Perlman [89] | 2014 | 17 | No | 9 | 8 | 4.5 | Community | Cross sectional | Neural mechanisms (i.e., lateral prefrontal cortex) sustaining frustration management during a task **↔** parent-reports of frustration tolerance in general |
| 55 | Quinones-Camacho [92] | 2020 | 117 | Yes | 63 | 54 | 4.9 | Community | Cross sectional | **↓** neural parent-child synchrony during frustration recovery **↔ ↑** child irritability |
| 56 | Ravi [56] | 2022 | 78 | Yes | NA | NA | 0.3 | Community | Longitudinal | Parents responded to their child’s negative emotions with distress/punishment, the child **→** **↑** irritability |
| 57 | Rohlf [69] | 2018 | 1501 | No | 728 | 773 | 8.4 | Community | Longitudinal | Partial mediation effect of irritability at age 9 in the relation between low EF at age 8 and physical aggression at age 11 |
| 58 | Ross [85] | 2021 | 40 | No | 20 | 20 | 15.7 | Clinical (Bipolar disorder) | Case-control | Functional connectivity abnormalities **↔** impact of frustrative non-reward and emotion dysregulation on decision making and attention |
| 59 | Salum [72] | 2017 | 1872 | No | 987 | 885 | 10.5 | Clinical (ADHD, CD, Anxiety) | Cross sectional | Irritability **↔↑** attention toward threat stimuli |
| 60 | Santana [91] | 2023 | 96 | No | 51 | 45 | 4.6 | Community | Cross sectional | Effortful control, but not irritability **↔** psychophysiological response of incongruent affect during frustration management among preschoolers |
| 61 | Scheinost [148] | 2021 | 69 | No | 38 | 31 | 14.6 | Clinical (DMDD, ADHD, Anxiety) | Cross sectional | Irritability **↔** functional connectivity during frustration management in adolescents |
| 62 | Silver [110] | 2022 | 541 | Yes | 293 | 248 | 3.6 | Community | Longitudinal | When measured at age 3, phasic but not tonic irritability **↔ ↓** effortful control and **↑** maladaptive parenting; only tonic irritability **→**  psychopathology (i.e., disruptive and suicidal behavior) |
| 63 | Silver [60] | 2022 | 452 | No | 250 | 202 | 6.1 | Clinical (ODD DBD, GAD, ADHD) | Longitudinal | Tonic irritability **→** internalizing disorders; Phasic irritability **→** externalizing disorders |
| 64 | Spangler [96] | 1993 | 42 | Yes | 24 | 18 | 0.0 | Community | Longitudinal | Focused mainly on a combination of both the ANS and HPA axis  In newborns, **↑** HR **↔**  **↑** irritability;  **↓** orientation (attentional skills) **↔ ↑** HRV; irritability ↛ cortisol regulation |
| 65 | Tseng [84] | 2019 | 195 | No | 98 | 97 | 12.9 | Clinical (DMDD, ADHD, Anxiety) | Cross sectional | After frustration management, irritability **↔** neural substrates of attention orienting (i.e., top-down regulation of emotion and motor execution) |
| 65 | Tseng [75] | 2021 |  |  |  |  |  |  |  | Aberrant amygdala-prefrontal-parietal circuitry during memory processes of threat stimuli (i.e., extinction recall) = common phenotype between irritability and anxiety |
| 66 | Ucar [43] | 2018 | 86 | Yes | 53 | 33 | 13.7 | Clinical (ADHD) | Case-control | ADHD + **↑** irritability **↔** **↑** negative parental attitudes |
| 67 | Valencia [107] | 2021 | 470 | Yes | 225 | 245 | 3.0 | Community | Longitudinal | Child irritability mediated the transmission of maternal internalizing symptoms to the child |
| 68 | van den Akker [106] | 2010 | 96 | Yes | 54 | 41 | 2.5 | Community | Longitudinal | **↑** anger proneness + activity ↔ **↓** positive parenting + **↑** externalizing symptoms;  **↑** anger proneness + social fear **↔ ↓** positive and **↑** negative parenting + **↑** internalizing problems |
| 69 | Vogel [149] | 2021 | 302 | No | 145 | 157 | 4.0 | Clinical (Depression) | Longitudinal | Irritability (i.e., dysregulation of anger or frustration) **→ ↓** later emotion dysregulation of negative emotion, whereas excitability (i.e., dysregulation of positive affect) remained stable |
| 69 | Vogel [77] | 2019 |  |  |  |  |  |  |  | ↑ irritability + **↓** regulation of positive emotions **→** psychopathology |
| 70 | von den Boom [99] | 1994 | 30 | Yes | 16 | 14 | 0.0 | Community | Longitudinal | Intervention for maternal sensitive responsiveness for mothers with highly irritable infants **→** **↑** quality of mother-infant interaction + **↑** child’s self-soothing abilities |
| 71 | Whedon [150] | 2021 | 160 | No | 86 | 74 | 0.4 | Community | Longitudinal | Children prone to experience anger + frustration: maturity of private speech during problem solving **→** development of inhibitory control and then emotion regulation |
| 72 | Zhang [73] | 2021 | 178 | No | 110 | 68 | 15.72 | Clinical (CD) | Case-control | **↑** callous-unemotional traits + **↑** irritability **↔** **↓** threat response; **↑** irritability alone **↔** **↑** response to threat |
| 72 | Zhang [45] | 2023 |  |  |  |  |  |  |  | CU traits but not irritability **↔** **↓** reinforcement-based decision-making |
| 73 | Zhang [57] | 2023 | 333 | No | 181 | 152 | 1.5 | Community | Longitudinal | **↑** effortful control **↔ ↓** irritability but did not predict an increase in irritability |
| 74 | Zik [80] | 2022 | 195 | No | 115 | 80 | 13.2 | Clinical (DMDD, ADHD) | Cross sectional | Chronic irritability **↔** anger expression but less to aggression and anger control |

**Note.** N: Number of participants in the analytic sample. Parents: are parents included in the study. Mean age in years. **↑ :** increase, more, higher, improvement; **↓:** decrease, less, smaller; **↔:** association (cross-sectional); ↮: no association (cross-sectional); **→ :** direction (longitudinal); ↛ : no prediction (longitudinal); Ո: inverted U association (nonlinear association); + : and, alongside; PFC: prefrontal cortex; dLPFC: dorsolateral prefrontal cortex; RSA: respiratory sinus arrhythmia; EF: executive functions; ADHD: attention deficits with or without hyperactivity disorder; DMDD: disruptive mood dysregulation disorder; ERN: error related negativity; ANS: autonomous nervous system; ODD: oppositional defiant disorder; HPA: hypothalamic–pituitary–adrenal; HR: heart rate; HRV: Heart rate variability; CU: Callous-unemotional; ADHD : Attention-deficit/hyperactivity disorder; CD : Conduct disorders; DBD: disruptive behavior disorders; DMDD : Disruptive mood dysregulation disorder; GAD,: generalized anxiety disorder; MDD: Major depressive disorder; ODD: Oppositional defiant disorder; PTSD: Post-Traumatic Stress Disorder; SAD : Seasonal Affective Disorder; SMDD: Severe Mood Dysregulation Disorder.

**Table S2.** Assessment of bias

| **Authors** | **Year** | **#1** | **#2** | **#3** | **#4** | **#5** | **#6** | **#7** | **#8** | **#9** | **#10** | **#11** | **#12** | **#13** | **#14** | **#15** | **#16** | **#17** | **#18** | **#19** | **#20** |
| --- | --- | --- | --- | --- | --- | --- | --- | --- | --- | --- | --- | --- | --- | --- | --- | --- | --- | --- | --- | --- | --- |
| Ali [90] | 2023 | Yes | Yes | No | Yes | Yes | Yes | Yes | Yes | No | Yes | Yes | Yes | No | Yes | Yes | Yes | Yes | Yes | No | Yes |
| Ametti [94] | 2022 | Yes | Yes | No | Yes | Yes | Yes | No | Yes | Yes | Yes | Yes | Yes | Unknown | NA | Yes | Yes | Yes | Yes | No | Yes |
| Armour [105] | 2018 | Yes | Yes | No | Yes | Yes | Yes | No | Yes | Yes | Yes | Yes | Yes | Unknown | NA | Yes | Yes | Yes | Yes | No | Yes |
| Barbosa [98] | 2019 | Yes | Yes | No | Yes | Yes | Yes | No | Yes | Yes | Yes | Yes | Yes | Unknown | NA | Yes | Yes | Yes | Yes | No | Yes |
| Blair [38] | 2020 | Yes | Yes | No | Yes | Yes | Yes | No | Yes | Yes | Yes | Yes | Yes | Unknown | NA | Yes | Yes | Yes | Yes | No | Yes |
| Braenden [144] | 2023 | Yes | Yes | No | Yes | Yes | Yes | Yes | Yes | Yes | Yes | Yes | Yes | No | No | Yes | Yes | Yes | Yes | No | Yes |
| Braenden [39] | 2023 | Yes | Yes | No | Yes | Yes | Yes | No | Yes | Yes | Yes | Yes | Yes | Unknown | NA | Yes | Yes | Yes | Yes | No | Yes |
| Calkins [104] | 2004 | Yes | Yes | No | Yes | Yes | Yes | Yes | Yes | Yes | Yes | Yes | Yes | No | No | Yes | Yes | Yes | Yes | No | Yes |
| Cardinale [65] | 2022 | Yes | Yes | No | Yes | Yes | Yes | No | Yes | Yes | Yes | Yes | Yes | Unknown | NA | Yes | Yes | Yes | Yes | No | Yes |
| Cardinale [44] | 2021 | Yes | Yes | No | Yes | Yes | Yes | No | Yes | Yes | Yes | Yes | Yes | Unknown | NA | Yes | Yes | Yes | Yes | No | Yes |
| Cave-Freeman [101] | 2023 | Yes | Yes | No | Yes | Yes | Yes | No | Yes | Yes | Yes | Yes | Yes | Unknown | NA | Yes | Yes | Yes | Yes | No | Yes |
| Cha [108] | 2018 | Yes | Yes | No | Yes | Yes | Yes | No | Yes | Yes | Yes | Yes | Yes | Unknown | NA | Yes | Yes | Yes | Yes | No | Yes |
| Chaarani [64] | 2017 | Yes | Yes | No | Yes | Yes | Yes | No | Yes | Yes | Yes | Yes | Yes | Unknown | NA | Yes | Yes | Yes | Yes | No | Yes |
| Colonna [47] | 2022 | Yes | Yes | No | Yes | Yes | Yes | No | Yes | Yes | Yes | Yes | Yes | Unknown | NA | Yes | Yes | Yes | Yes | No | Yes |
| Crockenberg [100] | 1981 | Yes | Yes | No | Yes | Yes | Yes | No | Yes | Yes | Yes | Yes | Yes | Unknown | NA | Yes | Yes | Yes | No | No | Yes |
| Crum [95] | 2021 | Yes | Yes | No | Yes | Yes | Yes | No | Yes | Yes | Yes | Yes | Yes | Unknown | NA | Yes | Yes | Yes | Yes | No | Yes |
| Derella [93] | 2018 | Yes | Yes | No | Yes | Yes | No | No | Yes | Yes | Yes | Yes | Yes | Yes | Yes | Yes | Yes | Yes | Yes | No | Yes |
| Deveney [82] | 2013 | Yes | Yes | No | Yes | Yes | Yes | No | Yes | Yes | Yes | Yes | Yes | Unknown | NA | Yes | Yes | Yes | Yes | No | Yes |
| Deveney [40] | 2020 | Yes | Yes | No | Yes | Yes | Yes | No | Yes | Yes | Yes | Yes | Yes | Yes | Yes | Yes | Yes | Yes | Yes | No | Yes |
| Di Giunta[102] | 2018 | Yes | Yes | No | Yes | Yes | Yes | No | Yes | Yes | Yes | Yes | Yes | Yes | Yes | Yes | Yes | Yes | Yes | No | Yes |
| Dougherty [49] | 2023 | Yes | Yes | No | Yes | Yes | Yes | No | Yes | Yes | Yes | Yes | Yes | Unknown | NA | Yes | Yes | Yes | Yes | No | Yes |
| Elvin [66] | 2021 | Yes | Yes | No | Yes | Yes | Yes | No | Yes | Yes | Yes | Yes | Yes | Unknown | NA | Yes | Yes | Yes | Yes | No | Yes |
| Evans [81] | 2020 | Yes | Yes | No | Yes | Yes | Yes | No | Yes | Yes | Yes | Yes | Yes | Unknown | NA | Yes | Yes | Yes | Yes | No | Yes |
| Ezpeleta [58] | 2019 | Yes | Yes | No | Yes | Yes | Yes | No | Yes | Yes | Yes | Yes | Yes | Unknown | NA | Yes | Yes | Yes | Yes | No | Yes |
| Filippi [63] | 2020 | Yes | Yes | No | Yes | Yes | Yes | No | Yes | Yes | Yes | Yes | Yes | Unknown | NA | Yes | Yes | Yes | Yes | No | Yes |
| Giller [71] | 2021 | Yes | Yes | No | Yes | Yes | Yes | No | Yes | Yes | Yes | Yes | Yes | Unknown | NA | Yes | Yes | Yes | Yes | No | Yes |
| Grabell [86] | 2018 | Yes | Yes | No | Yes | Yes | Yes | No | Yes | Yes | Yes | Yes | Yes | Unknown | NA | Yes | Yes | Yes | Yes | No | Yes |
| Grabell [41] | 2022 | Yes | Yes | No | Yes | Yes | Yes | No | Yes | Yes | Yes | Yes | Yes | No | No | Yes | Yes | Yes | yes | No | Yes |
| Gunning [97] | 2013 | Yes | Yes | No | Yes | Yes | Yes | No | Yes | Yes | Yes | Yes | Yes | Unknown | NA | Yes | Yes | Yes | Yes | No | Yes |
| Haller [42] | 2021 | Yes | Yes | No | Yes | Yes | Yes | No | Yes | Yes | Yes | Yes | Yes | Unknown | NA | Yes | Yes | Yes | Yes | No | Yes |
| Harle [88] | 2022 | Yes | Yes | No | Yes | Yes | Yes | No | Yes | Yes | Yes | Yes | Yes | Unknown | NA | Yes | Yes | Yes | Yes | No | Yes |
| Hodgdon [83] | 2021 | Yes | Yes | No | Yes | Yes | Yes | No | Yes | Yes | Yes | Yes | Yes | Unknown | NA | Yes | Yes | Yes | Yes | No | Yes |
| Karalunas, [67] | 2021 | Yes | Yes | No | Yes | Yes | Yes | No | Yes | Yes | Yes | Yes | Yes | Unknown | NA | Yes | Yes | Yes | No | No | Yes |
| Karalunas [68] | 2023 | Yes | Yes | No | Yes | Yes | Yes | No | Yes | Yes | Yes | Yes | Yes | Unknown | NA | Yes | Yes | Yes | Yes | No | Yes |
| Karim [79] | 2017 | Yes | Yes | No | Yes | Yes | Yes | No | Yes | Yes | Yes | Yes | Yes | Unknown | NA | Yes | Yes | Yes | Yes | No | Yes |
| Kessel [53] | 2021 | Yes | Yes | No | Yes | Yes | Yes | No | Yes | Yes | Yes | Yes | Yes | Unknown | NA | Yes | Yes | Yes | Yes | No | Yes |
| Kessel [54] | 2016 | Yes | Yes | No | Yes | Yes | Yes | No | Yes | Yes | Yes | Yes | Yes | Unknown | NA | Yes | Yes | Yes | Yes | No | Yes |
| Kryza-Lacombe [76] | 2020 | Yes | Yes | No | Yes | Yes | Yes | No | Yes | Yes | Yes | Yes | Yes | Unknown | NA | Yes | Yes | Yes | Yes | No | Yes |
| Kryza-Lacombe [145] | 2020 | Yes | Yes | No | Yes | Yes | Yes | No | Yes | Yes | Yes | Yes | Yes | Unknown | NA | Yes | Yes | Yes | Yes | No | Yes |
| Kryza-Lacombe [74] | 2022 | Yes | Yes | No | Yes | Yes | Yes | No | Yes | Yes | Yes | Yes | Yes | Unknown | NA | Yes | Yes | Yes | Yes | No | Yes |
| Lee [61] | 2022 | Yes | Yes | No | Yes | Yes | Yes | Yes | Yes | Yes | Yes | Yes | Yes | No | Yes | Yes | Yes | Yes | Yes | No | Yes |
| Lee [103] | 2013 | Yes | Yes | No | Yes | Yes | Yes | Yes | Yes | Yes | Yes | Yes | Yes | No | Yes | Yes | Yes | Yes | Yes | No | Yes |
| Legenbauer [50] | 2018 | Yes | Yes | No | Yes | Yes | Yes | No | Yes | Yes | Yes | Yes | Yes | Unknown | NA | Yes | Yes | Yes | Yes | No | Yes |
| Leigh [78] | 2006 | Yes | Yes | No | Yes | Yes | Yes | Yes | Yes | Yes | Yes | Yes | Yes | No | Yes | Yes | Yes | Yes | Yes | No | Yes |
| Lengua [111] | 2006 | Yes | Yes | No | Yes | Yes | Yes | Yes | Yes | Yes | Yes | Yes | Yes | No | Yes | Yes | Yes | Yes | Yes | No | Yes |
| Lengua [109] | 2003 | Yes | Yes | No | Yes | Yes | Yes | Yes | Yes | Yes | Yes | Yes | Yes | Yes | No | Yes | Yes | Yes | Yes | No | Yes |
| Li [62] | 2017 | Yes | Yes | No | Yes | Yes | Yes | No | Yes | Yes | Yes | Yes | Yes | Unknown | NA | Yes | Yes | Yes | Yes | No | Yes |
| Linke [87] | 2023 | Yes | Yes | No | Yes | Yes | Yes | No | Yes | Yes | Yes | Yes | Yes | Unknown | NA | Yes | Yes | Yes | Yes | No | Yes |
| Liuzzi [51] | 2020 | Yes | Yes | No | Yes | Yes | Yes | No | Yes | Yes | Yes | Yes | Yes | Unknown | NA | Yes | Yes | Yes | Yes | No | Yes |
| Malhi [52] | 2021 | Yes | Yes | No | Yes | Yes | Yes | No | Yes | Yes | Yes | Yes | Yes | Unknown | NA | Yes | Yes | Yes | Yes | No | Yes |
| Morris [146] | 2002 | Yes | Yes | No | Yes | Yes | Yes | No | Yes | Yes | Yes | Yes | Yes | Unknown | NA | Yes | Yes | Yes | Yes | No | Yes |
| Naim [46] | 2021 | Yes | Yes | No | Yes | Yes | Yes | No | Yes | Yes | Yes | Yes | Yes | Unknown | NA | Yes | Yes | Yes | Yes | No | Yes |
| Naim [70] | 2022 | Yes | Yes | No | Yes | Yes | Yes | No | Yes | Yes | Yes | Yes | Yes | Unknown | NA | Yes | Yes | Yes | Yes | No | Yes |
| Nili [31] | 2022 | Yes | Yes | No | Yes | Yes | Yes | Yes | Yes | Yes | Yes | Yes | Yes | Yes | No | Yes | Yes | Yes | Yes | No | Yes |
| Nobakht [55] | 2023 | Yes | Yes | No | Yes | Yes | Yes | No | Yes | Yes | Yes | Yes | Yes | Yes | No | Yes | Yes | Yes | Yes | No | Yes |
| Perez [48] | 2021 | Yes | Yes | No | Yes | Yes | Yes | No | Yes | Yes | Yes | Yes | Yes | Unknown | NA | Yes | Yes | Yes | Yes | No | Yes |
| Perhamus [59] | 2021 | Yes | Yes | No | Yes | Yes | Yes | No | Yes | Yes | Yes | Yes | Yes | Unknown | NA | Yes | Yes | Yes | Yes | No | Yes |
| Perlman [147] | 2015 | Yes | Yes | No | Yes | Yes | Yes | No | Yes | Yes | Yes | Yes | Yes | Unknown | NA | Yes | Yes | Yes | Yes | No | Yes |
| Perlman [89] | 2014 | Yes | Yes | No | Yes | Yes | Yes | No | Yes | Yes | Yes | Yes | Yes | Unknown | NA | Yes | Yes | Yes | Yes | No | Yes |
| Quinones-Camacho [92] | 2020 | Yes | Yes | No | Yes | Yes | Yes | No | Yes | Yes | Yes | Yes | Yes | Unknown | NA | Yes | Yes | Yes | Yes | No | Yes |
| Ravi [56] | 2022 | Yes | Yes | No | Yes | Yes | Yes | No | Yes | Yes | Yes | Yes | Yes | Unknown | NA | Yes | Yes | Yes | Yes | No | Yes |
| Rohlf [69] | 2018 | Yes | Yes | No | Yes | Yes | Yes | No | Yes | Yes | Yes | Yes | Yes | Unknown | NA | Yes | Yes | Yes | Yes | No | Yes |
| Ross [85] | 2021 | Yes | Yes | No | Yes | Yes | Yes | No | Yes | Yes | Yes | Yes | Yes | Unknown | NA | Yes | Yes | Yes | Yes | No | Yes |
| Salum [72] | 2017 | Yes | Yes | No | Yes | Yes | Yes | Yes | Yes | Yes | Yes | Yes | Yes | No | Yes | Yes | Yes | Yes | Yes | No | Yes |
| Santana [91] | 2023 | Yes | Yes | Yes | Yes | Yes | Yes | No | Yes | Yes | Yes | Yes | Yes | Unknown | NA | Yes | Yes | Yes | Yes | No | Yes |
| Scheinost [148] | 2021 | Yes | Yes | No | Yes | Yes | Yes | No | Yes | Yes | Yes | Yes | Yes | Unknown | NA | Yes | Yes | Yes | Yes | No | Yes |
| Silver [110] | 2023 | Yes | Yes | No | Yes | Yes | Yes | No | Yes | Yes | Yes | Yes | Yes | Unknown | NA | Yes | Yes | Yes | Yes | No | Yes |
| Silver [60] | 2023 | Yes | Yes | No | Yes | Yes | Yes | No | Yes | Yes | Yes | Yes | Yes | Unknown | NA | Yes | Yes | Yes | Yes | No | Yes |
| Spangler [96] | 1993 | Yes | Yes | No | Yes | Yes | Yes | No | Yes | Yes | Yes | Yes | Yes | Unknown | NA | Yes | Yes | Yes | No | No | Yes |
| Tseng [84] | 2019 | Yes | Yes | No | Yes | Yes | Yes | No | Yes | Yes | Yes | Yes | Yes | Unknown | NA | Yes | Yes | Yes | Yes | No | Yes |
| Tseng [75] | 2021 | Yes | Yes | No | Yes | Yes | Yes | No | Yes | Yes | Yes | Yes | Yes | Unknown | NA | Yes | Yes | Yes | Yes | No | Yes |
| Ucar [43] | 2018 | Yes | Yes | Yes | Yes | Yes | Yes | No | Yes | Yes | Yes | Yes | Yes | Unknown | NA | Yes | Yes | Yes | Yes | No | Yes |
| Valencia [107] | 2021 | Yes | Yes | No | Yes | Yes | Yes | Yes | Yes | Yes | Yes | Yes | Yes | Yes | Yes | Yes | Yes | Yes | Yes | No | Yes |
| van den Akker [106] | 2010 | Yes | Yes | No | Yes | Yes | Yes | Yes | Yes | Yes | Yes | Yes | Yes | No | Yes | Yes | Yes | Yes | Yes | No | Yes |
| Vogel [149] | 2021 | Yes | Yes | No | Yes | Yes | Yes | No | Yes | Yes | Yes | Yes | Yes | Unknown | NA | Yes | Yes | Yes | Yes | No | Yes |
| Vogel [77] | 2019 | Yes | Yes | No | Yes | Yes | Yes | No | Yes | Yes | Yes | Yes | Yes | Unknown | NA | Yes | Yes | Yes | No | No | Yes |
| von den Boom [99] | 1994 | Yes | Yes | No | Yes | Yes | Yes | No | Yes | Yes | Yes | Yes | Yes | Unknown | NA | Yes | Yes | Yes | Yes | No | Yes |
| Whedon [150] | 2021 | Yes | Yes | No | Yes | Yes | Yes | Yes | Yes | Yes | Yes | Yes | Yes | No | Yes | Yes | Yes | Yes | Yes | No | Yes |
| Zhang [73] | 2021 | Yes | Yes | No | Yes | Yes | Yes | No | Yes | Yes | Yes | Yes | Yes | Unknown | NA | Yes | Yes | Yes | Yes | No | Yes |
| Zhang [45] | 2021 | Yes | Yes | No | Yes | Yes | Yes | No | Yes | Yes | Yes | Yes | Yes | Unknown | NA | Yes | Yes | Yes | Yes | No | Yes |
| Zhang [57] | 2023 | Yes | Yes | No | Yes | Yes | Yes | No | Yes | Yes | Yes | Yes | Yes | Unknown | NA | Yes | Yes | Yes | Yes | No | Yes |
| Zik [80] | 2022 | Yes | Yes | No | Yes | Yes | Yes | No | Yes | Yes | Yes | Yes | Yes | Unknown | NA | Yes | Yes | Yes | Yes | No | Yes |

**Note.** NA: Not applicable. The list of items: Introduction: #1: Were the aims/objectives of the study clear?; Methods: #2: Was the study design appropriate for the stated aim(s)?; #3: Was the sample size justified?; #4: Was the target/reference population clearly defined? (Is it clear who the research was about?); #5: Was the sample frame taken from an appropriate population base so that it closely represented the target/reference population under investigation?; #6: Was the selection process likely to select subjects/participants that were representative of the target/reference population under investigation?; #7: Were measures undertaken to address and categorize non-responders?; #8: Were the risk factor and outcome variables measured appropriate to the aims of the study?; #9: Were the risk factor and outcome variables measured correctly using instruments/measurements that had been trialed, piloted or published previously?; #10: Is it clear what was used to determined statistical significance and/or precision estimates? (e.g., p values, CIs); #11: Were the methods (including statistical methods) sufficiently described to enable them to be repeated?; Results: #12: Were the basic data adequately described?; #13: Does the response rate raise concerns about non-response bias?; #14: If appropriate, was information about non-responders described?; #15: Were the results internally consistent?; #16: Were the results for the analyses described in the methods, presented?; Discussion: #17: Were the authors’ discussions and conclusions justified by the results?; #18: Were the limitations of the study discussed? Other: #19: Were there any funding sources or conflicts of interest that may affect the authors’ interpretation of the results? #20: Was ethical approval or consent of participants attained?

**Figure S2.** Summary of the AXIS assessment

**Note.** NA: Not applicable. The list of items: Introduction: #1: Were the aims/objectives of the study clear?; Methods: #2: Was the study design appropriate for the stated aim(s)?; #3: Was the sample size justified?; #4: Was the target/reference population clearly defined? (Is it clear who the research was about?); #5: Was the sample frame taken from an appropriate population base so that it closely represented the target/reference population under investigation?; #6: Was the selection process likely to select subjects/participants that were representative of the target/reference population under investigation?; #7: Were measures undertaken to address and categorize non-responders?; #8: Were the risk factor and outcome variables measured appropriate to the aims of the study?; #9: Were the risk factor and outcome variables measured correctly using instruments/measurements that had been trialed, piloted or published previously?; #10: Is it clear what was used to determined statistical significance and/or precision estimates? (e.g., p values, CIs); #11: Were the methods (including statistical methods) sufficiently described to enable them to be repeated?; Results: #12: Were the basic data adequately described?; #13: Does the response rate raise concerns about non-response bias?; #14: If appropriate, was information about non-responders described?; #15: Were the results internally consistent?; #16: Were the results for the analyses described in the methods, presented?; Discussion: #17: Were the authors’ discussions and conclusions justified by the results?; #18: Were the limitations of the study discussed? Other: #19: Were there any funding sources or conflicts of interest that may affect the authors’ interpretation of the results? #20: Was ethical approval or consent of participants attained?

Supplementary file 1 - Bibliographic database search strategies

**Understanding irritability through the lens of self-regulatory control processes in children and adolescents: A systematic review**

*Sébastien Urben, Ana Ochoa Williams, Cécile Ben Jemia, Joëlle Rosselet Amoussou, Sara Machado Lazaro, Julia Giovannini, Marion Abi Kheir, Michael Kaess, Kerstin von Plessen & Ines Mürner-Lavanchy*

Bibliographic database search strategies

The research strategies were peer reviewed by another information specialist prior to execution.

**Embase.com**

1121 references found, 12 October 2023

('self control'/exp OR 'emotion dysregulation'/exp OR 'emotional control'/exp OR 'difficulties in emotion regulation scale'/exp OR 'affective reactivity index'/exp OR 'heart rate'/de OR 'heart rate variability'/de OR 'respiratory sinus arrhythmia'/de OR 'vagus tone'/de OR 'pressoreceptor reflex'/de OR 'autonomic nervous system'/de OR 'adrenergic system'/de OR 'executive function'/de OR 'metacognition'/exp OR 'ego'/de OR (((emotion* OR anger OR frustration OR affective OR affect) NEAR/3 (control OR regulat* OR dysregulat*)) OR self-control OR "self manag*" OR self-regulat* OR "emotional restraint" OR "emotional adjustment*" OR "Self regulatory" OR "Affective Reactivity Index" OR "36-item DERS" OR "DERS scale" OR ((cardiac OR heart) NEXT/1 (frequenc* OR rate*)) OR "respiratory sinus arrhythmia" OR "vagus tone" OR "vagal tone" OR vagotonus OR "vagus nerve tone" OR baroreflex* OR "pressoreceptor reflex" OR "baroceptor reflex" OR "baroreceptor reflex" OR "pressor reflex" OR "pressure reflex" OR ((autonom* OR vegetative) NEXT/3 system) OR ((adrenergic OR sympath* OR orthosympath*) NEXT/3 (mechanism OR system)) OR "executive function*" OR "cognitive control" OR ((executive OR proactive OR reactive) NEXT/1 control) OR "effortful control" OR metacognition OR metacognitive OR meta-cognit* OR "ego control" OR "ego undercontrol" OR "ego resiliency" OR "ego depletion"):ab,ti,kw) AND ('irritability'/exp OR (irritability OR (irritable NOT "irritable bowel")):ab,ti,kw) AND ('child'/exp OR 'adolescent'/exp OR 'adolescence'/exp OR 'high school student'/exp OR 'elementary student'/exp OR 'middle school student'/exp OR 'juvenile'/de OR (child* OR adolescen* OR preadolescen* OR pre-adol* OR teen* OR youth* OR "high school" OR "elementary school" OR "elementary student*" OR "middle school" OR preschool* OR juvenile):ab,ti,kw)

**Medline ALL Ovid**

Ovid MEDLINE(R) ALL 1946 to October 11, 2023

438 references found, 12 October 2023

(exp Self-Control/ OR Heart Rate/ OR Respiratory Sinus Arrhythmia/ OR Autonomic Nervous System/ OR Executive Function/ OR Metacognition/ OR Ego/ OR (((emotion* OR anger OR frustration OR affective OR affect) ADJ3 (control OR regulat* OR dysregulat*)) OR self-control OR "self manag*" OR self-regulat* OR "emotional restraint" OR "emotional adjustment*" OR "Self regulatory" OR "Affective Reactivity Index" OR "36-item DERS" OR "DERS scale" OR ((cardiac OR heart) ADJ1 (frequenc* OR rate*)) OR "respiratory sinus arrhythmia" OR "vagus tone" OR "vagal tone" OR vagotonus OR "vagus nerve tone" OR baroreflex* OR "pressoreceptor reflex" OR "baroceptor reflex" OR "baroreceptor reflex" OR "pressor reflex" OR "pressure reflex" OR ((autonom* OR vegetative) ADJ3 system) OR ((adrenergic OR sympath* OR orthosympath*) ADJ3 (mechanism OR system)) OR "executive function*" OR "cognitive control" OR ((executive OR proactive OR reactive) ADJ1 control) OR "effortful control" OR metacognition OR metacognitive OR meta-cognit* OR "ego control" OR "ego undercontrol" OR "ego resiliency" OR "ego depletion").ab,ti,kf.) AND ("Irritable Mood"/ OR (irritability OR (irritable NOT "irritable bowel")).ab,ti,kf.) AND (exp Child/ OR Adolescent/ OR (child* OR adolescen* OR preadolescen* OR pre-adol* OR teen* OR youth* OR "high school" OR "elementary school" OR "elementary student*" OR "middle school" OR preschool* OR juvenile).ab,ti,kf.)

**APA PsycInfo Ovid**

APA PsycInfo 1806 to October Week 1 2023

530 references found, 12 October 2023

(exp self-control/ OR emotional regulation/ OR exp emotional control/ OR exp heart rate/ OR autonomic nervous system/ OR exp executive function/ OR metacognition/ OR Ego/ OR (((emotion* OR anger OR frustration OR affective OR affect) ADJ3 (control OR regulat* OR dysregulat*)) OR self-control OR "self manag*" OR self-regulat* OR "emotional restraint" OR "emotional adjustment*" OR "Self regulatory" OR "Affective Reactivity Index" OR "36-item DERS" OR "DERS scale" OR ((cardiac OR heart) ADJ1 (frequenc* OR rate*)) OR "respiratory sinus arrhythmia" OR "vagus tone" OR "vagal tone" OR vagotonus OR "vagus nerve tone" OR baroreflex* OR "pressoreceptor reflex" OR "baroceptor reflex" OR "baroreceptor reflex" OR "pressor reflex" OR "pressure reflex" OR ((autonom* OR vegetative) ADJ3 system) OR ((adrenergic OR sympath* OR orthosympath*) ADJ3 (mechanism OR system)) OR "executive function*" OR "cognitive control" OR ((executive OR proactive OR reactive) ADJ1 control) OR "effortful control" OR metacognition OR metacognitive OR meta-cognit* OR "ego control" OR "ego undercontrol" OR "ego resiliency" OR "ego depletion").mp.) AND (irritability/ OR (irritability OR (irritable NOT "irritable bowel")).mp.) AND (child* OR adolescen* OR preadolescen* OR pre-adol* OR teen* OR youth* OR "high school" OR "elementary school" OR "elementary student*" OR "middle school" OR preschool* OR juvenile).mp.

**Cochrane Database of Systematic Reviews Wiley**

Cochrane Database of Systematic Reviews, Issue 10 of 12, October 2023

0 references found, 12 October 2023

(((emotion* OR anger OR frustration OR affective OR affect) NEAR/3 (control OR regulat* OR dysregulat*)) OR self-control OR (self NEXT manag*) OR self-regulat* OR "emotional restraint" OR (emotional NEXT adjustment*) OR "Self regulatory" OR "Affective Reactivity Index" OR "36-item DERS" OR "DERS scale" OR ((cardiac OR heart) NEXT/1 (frequenc* OR rate*)) OR "respiratory sinus arrhythmia" OR "vagus tone" OR "vagal tone" OR vagotonus OR "vagus nerve tone" OR baroreflex* OR "pressoreceptor reflex" OR "baroceptor reflex" OR "baroreceptor reflex" OR "pressor reflex" OR "pressure reflex" OR ((autonom* OR vegetative) NEXT/3 system) OR ((adrenergic OR sympath* OR orthosympath*) NEXT/3 (mechanism OR system)) OR (executive NEXT function*) OR "cognitive control" OR ((executive OR proactive OR reactive) NEXT/1 control) OR "effortful control" OR metacognition OR metacognitive OR meta-cognit* OR "ego control" OR "ego undercontrol" OR "ego resiliency" OR "ego depletion"):ab,ti,kw AND (irritability OR (irritable NOT "irritable bowel")):ab,ti,kw AND (child* OR adolescen* OR preadolescen* OR pre-adol* OR teen* OR youth* OR "high school" OR "elementary school" OR (elementary NEXT student*) OR "middle school" OR preschool* OR juvenile):ab,ti,kw

**Web of Science Core collection**

Science Citation Index Expanded (1900-present), Social Sciences Citation Index (1900-present), Arts & Humanities Citation Index (1975-present), Conference Proceedings Citation Index-Science (1990-present), Book Citation Index (2005-present), Emerging Sources Citation Index (2005-present), Current Chemical Reactions and Index Chemicus

Advanced search > More options > Exact search

497 references found, 12 October 2023

TS=((((emotion* OR anger OR frustration OR affective OR affect) NEAR/2 (control OR regulat* OR dysregulat*)) OR self-control OR "self manag*" OR self-regulat* OR "emotional restraint" OR "emotional adjustment*" OR "Self regulatory" OR "Affective Reactivity Index" OR "36-item DERS" OR "DERS scale" OR ((cardiac OR heart) NEAR/1 (frequenc* OR rate*)) OR "respiratory sinus arrhythmia" OR "vagus tone" OR "vagal tone" OR vagotonus OR "vagus nerve tone" OR baroreflex* OR "pressoreceptor reflex" OR "baroceptor reflex" OR "baroreceptor reflex" OR "pressor reflex" OR "pressure reflex" OR ((autonom* OR vegetative) NEAR/2 system) OR ((adrenergic OR sympath* OR orthosympath*) NEAR/2 (mechanism OR system)) OR "executive function*" OR "cognitive control" OR ((executive OR proactive OR reactive) NEAR/1 control) OR "effortful control" OR metacognition OR metacognitive OR meta-cognit* OR "ego control" OR "ego undercontrol" OR "ego resiliency" OR "ego depletion") AND (irritability OR (irritable NOT "irritable bowel")) AND (child* OR adolescen* OR preadolescen* OR pre-adol* OR teen* OR youth* OR "high school" OR "elementary school" OR "elementary student*" OR "middle school" OR preschool* OR juvenile))

**ProQuest Dissertations & Theses A&I**

Doctoral dissertations only

Search field : NOFT

26 references found, 12 October 2023

(((emotion* OR anger OR frustration OR affective OR affect) NEAR/2 (control OR regulat* OR dysregulat*)) OR self-control OR self-regulat* OR "emotional restraint" OR "emotional adjustment" OR "Self regulatory" OR "Affective Reactivity Index" OR "36-item DERS" OR "DERS scale" OR ((cardiac OR heart) PRE/0 (frequenc* OR rate*)) OR "respiratory sinus arrhythmia" OR "vagus tone" OR "vagal tone" OR vagotonus OR "vagus nerve tone" OR baroreflex* OR "pressoreceptor reflex" OR "baroceptor reflex" OR "baroreceptor reflex" OR "pressor reflex" OR "pressure reflex" OR ((autonom* OR vegetative) PRE/2 system) OR ((adrenergic OR sympath* OR orthosympath*) PRE/2 (mechanism OR system)) OR "executive function" OR "cognitive control" OR ((executive OR proactive OR reactive) PRE/0 control) OR "effortful control" OR metacognition OR metacognitive OR meta-cognit* OR "ego control" OR "ego undercontrol" OR "ego resiliency" OR "ego depletion") AND irritability AND (child* OR adolescen* OR youth*)
